# Retrospective comparative analysis of human fascioliasis versus bacterial liver abscess

**DOI:** 10.1097/MD.0000000000040914

**Published:** 2024-12-13

**Authors:** Lihua Huang, Jiao Luo, Nan Li, Zhenzhong Bao, Wei Gu

**Affiliations:** a Department of Infection Disease, The First Affiliated Hospital of Dali University, Dali, Yunnan, PR China.

**Keywords:** bacterial liver abscess, clinical characteristics, epidemiological features, human fascioliasis

## Abstract

This study aimed to compare the clinical characteristics of human fascioliasis and bacterial liver abscesses to provide a reference for the early and effective differentiation of these 2 diseases to avoid misdiagnosis. We retrospectively compared the epidemiological and clinical characteristics of 95 patients with human fascioliasis and 95 with bacterial liver abscess admitted to the First Affiliated Hospital of Dali University between January 2013 and March 30, 2023. The human fascioliasis group exhibited a higher proportion of female patients and a greater frequency of abdominal pain and lesions affecting both lobes of the liver. Additionally, eosinophil count, percentage of eosinophils, red blood cells, hemoglobin, total protein, albumin, carbohydrate antigen-125 (CA-125), CA-199, and CA-724 levels were elevated compared to the bacterial liver abscess group. Conversely, the proportion of patients with diabetes, duration of hospital stay, and the incidence of lesions affecting the right lobe of the liver, cavity formation, ascites, pleural effusion, white blood cells, total bilirubin, alkaline phosphatase, alanine aminotransferase, aspartate aminotransferase, and gamma-glutamyl transferase levels were lower in the fascioliasis group compared to the bacterial liver abscess group. Furthermore, higher thrombin time and fibrinogen levels were observed in the fascioliasis group than in the bacterial liver abscess group (*P < *.05). Human fascioliasis predominantly occurs from August to November, while bacterial liver abscess exhibits a consistent incidence throughout the year. Human fascioliasis predominantly affects young women and demonstrates a seasonal pattern. It is associated with severe abdominal symptoms, widespread lesions, significantly elevated eosinophil levels, and seasonal incidence. Bacterial liver abscess predominantly affects elderly men with diabetes, and it is associated with localized lesions and severe inflammatory manifestations and occurs throughout the year.

## 1. Introduction

Human fascioliasis and bacterial liver abscess, both of which affect the liver through different etiologies, present a significant health risk globally, particularly among patients with diabetes, the elderly, and individuals with specific lifestyle habits, such as those consuming raw water products or utilizing untreated water sources. This has significantly increased the prevalence of these 2 conditions.^[1–3]^ Fascioliasis is a parasitic disease caused by *Fasciola hepatica* or *Fasciola gigantic,* while bacterial liver abscess arises primarily from the invasion of liver tissue by various pathogenic bacteria. The clinical manifestations of these 2 conditions exhibit similarities, thereby complicating the diagnosis.^[4,5]^

Accurate and prompt distinction between human fascioliasis and bacterial liver abscesses is essential for determining appropriate treatment protocols. Significant differences exist in their pathogenesis, pathological processes, and therapeutic options. Misdiagnosis can lead to ineffective treatment, exacerbation of symptoms, and delayed intervention for critical complications.^[5–7]^ Furthermore, a comprehensive understanding of their distinct epidemiological features can assist public health institutions in implementing more focused preventive measures.

Despite advancements in studying human fascioliasis and bacterial liver abscesses, a systematic and comprehensive comparison of their essential characteristics, imaging findings, and laboratory results remains limited.^[5,6]^ We need to further investigate and enhance our understanding of their seasonal distribution patterns and potential influencing factors.

This study involved a comprehensive case-control analysis comparing the clinical characteristics, comorbidities, hospitalization durations, imaging findings, and laboratory results of 95 patients with human fascioliasis to 95 patients with bacterial liver abscess. Moreover, this study aimed to clarify the differences in seasonal onset patterns between the 2 patient groups and identify potential environmental risk factors. This comprehensive data analysis aimed to provide clinicians with a more definitive basis for differential diagnosis and offer scientific support for enhancing public health prevention and control measures.

## 2. Materials and methods

### 2.1. Ethics approval and consent to participate

The study was approved by the Medical Ethics Committee of the First Affiliated Hospital of Dali University. The need for informed consent was waived due to the retrospective nature of this study.

### 2.2. Study population

This study utilized a retrospective cohort design to collect and analyze clinical data from 95 patients with human fascioliasis, matched with 95 patients with bacterial liver abscess during the same period. The diagnostic criteria for human fascioliasis strictly followed the Health Industry Standard of the People’s Republic of China (WS/T566-2017).^[8]^ The diagnosis of the bacterial liver abscess was based on the Expert Consensus on the Emergency Diagnosis and Treatment of Bacterial Liver Abscess, published in 2022. This consensus primarily includes a positive bacterial culture from liver abscess aspiration or confirmation of liver abscess through abdominal ultrasound, computed tomography (CT), magnetic resonance imaging (MRI), and other relevant examinations, with a positive response to antibacterial treatment.^[9]^ Patients with tuberculous or amebic liver abscess who demonstrated compromised immune function or patients with incomplete clinical data were excluded from the human fascioliasis and bacterial liver abscess groups.

### 2.3. Sample selection and patient information collection

The demographic characteristics of each patient, including age and gender, were carefully recorded. The median and interquartile range were calculated and displayed to illustrate the age distribution. Additionally, a systematic screening was performed to evaluate the prevalence of underlying diseases, including diabetes, biliary stones, and hypertension. Vital signs were systematically recorded during hospitalization, including temperature, pulse, respiratory rate, and blood pressure. A comprehensive analysis was performed to investigate the frequency of various clinical manifestations, including fever, abdominal pain, nausea, vomiting, and respiratory symptoms. Besides, the study examined the variation in hospitalization duration among the patients.

### 2.4. Imaging data acquisition and analysis

Thoracic or abdominal CT scans were used to comprehensively examine the hepatic condition of patients. Comprehensive records were compiled based on the number, location, and specific imaging features of each observed lesion. Furthermore, the ratios of solitary and multiple lesions and the asymmetric distribution of lesions between the left and right lobes of the liver were recorded. Additionally, careful observation and comparison were performed to identify and record the incidence and prevalence of complications, including gas cavities, ascites, and pleural effusions. Particular attention was directed towards identifying splenomegaly due to its significant clinical importance.

### 2.5. Collection of laboratory indicators

Blood routine testing was performed to obtain crucial indicators, including white blood cells (WBC), red blood cells (RBC), hemoglobin (HGB), and platelet counts. We primarily obtained the assessments of total bilirubin (TBI), alanine aminotransferase (ALT), aspartate aminotransferase (AST), alkaline phosphatase ALP, gamma-glutamyl transferase (GGT), total protein (TP), albumin (ALB), and globulin levels to assess liver function. Furthermore, we collected data on coagulation factors, including prothrombin time (PT), activated partial thromboplastin time, thrombin time, and fibrinogen (FIB) levels. We categorize the time of disease onset in each case based on the month to understand the seasonal patterns of disease occurrence. This information was subsequently utilized to create a seasonal distribution map of disease onset, visually representing the seasonal variations in disease occurrence.

### 2.6. Statistical analysis methods and processes

Statistical Package for the Social Sciences software (version 26.0; IBM Corp., Armonk) was used for data analysis. Counting data are presented as a percentage (%), and the χ² test or the Fisher exact probability method was used for the group comparison. Measurement data adhering to the normal distribution are expressed as mean ± standard deviation, and the independent sample *t*-test was employed for group comparisons. However, measurement data not conforming to the normal distribution are represented by M (Q1-3), and the Mann–Whitney *U* test was used for group comparisons. The significance level was set at α = 0.05, indicating that *P *< .05 was considered statistically significant.

## 3. Results

### 3.1. Comparison of baseline data and clinical characteristics of patients

Regarding age, the mean age of patients in the human fascioliasis group was 40.00 (27.00–51.00), significantly younger than that of patients in the bacterial liver abscess group 57.00 (48.00–66.00; *P < *.05). Regarding sex, 38.95% of patients in the human fascioliasis group were male, while 56.84% of patients in the bacterial liver abscess group were males (*P < *.05). Regarding comorbid conditions, 5.26% and 2.11% of patients in the human fascioliasis group had diabetes and biliary stones, respectively, while 26.32% and 10.53% of patients in the bacterial liver abscess had diabetes and biliary stones, respectively (*P < *.05). No significant difference was observed between the percentage of patients with hypertension between the 2 groups. Regarding vital signs, patients in the human fascioliasis group exhibited a systolic blood pressure of 116.00 (106.00–131.00) and a diastolic blood pressure of 75.44 ± 9.61, while patients in the bacterial liver abscess group exhibited a systolic blood pressure of 111.00 (97.00–124.00) and a diastolic blood pressure of 70.54 ± 13.78 (*P < *.05). Regarding hospitalization duration, patients in the human fascioliasis group exhibited significantly longer days of hospitalization than those in the bacterial liver abscess group (7.00 [5.00–12.00] vs 12.00 [8.00–17.00]) (Table 1).

**Table 1:** Clinical data of patients with human fascioliasis and bacterial liver abscess.

Variable	Human fascioliasis (n = 95)	Bacterial liver abscess (n = 95)	χ^2^/t/z	*P*-value
Patient characteristics				
Age (yr [median, IQR])	40.00 (27.00–51.00)	57.00 (48.00–66.00)	−7.26	<.001
Sex, male (n, %)	37 (38.95%)	54 (56.84%)	6.095	.014
Body temperature (°C)	36.60 (36.30–37.70)	36.60 (36.50–37.00)	−0.07	.943
Pulse rate (beast/min)	92.00 (82.00–102.00)	92.00 (81.00–101.00)	−0.073	.942
Breath rate (beast/min)	20.00 (20.00–21.00)	20.00 (20.00–20.00)	−0.014	.989
Comorbidities (n, %)				
Hypertension	14 (14.74%)	24 (25.26%)	3.289	.070
Diabetes	5 (5.26%)	25 (26.32%)	15.833	<.001
Biliary tract stone	2 (2.11%)	10 (10.53%)	5.693	.017
Clinical symptoms (n, %)				
Fever	37 (38.95%)	43 (45.26%)	0.777	.378
Abdominal pain	67 (70.53%)	18 (18.95%)	51.114	<.001
Nausea and vomiting	2 (2.11%)	6 (6.32%)	1.174	.278
Lower respiratory symptoms	6 (6.32%)	3 (3.16%)	0.467	.495
Hospital stays (d [median, IQR])	7.00 (5.00–12.00)	12.00 (8.00–17.00)	−4.370	<.001

The baseline data used in this study were consistent with data in our previously published study.^[10]^

### 3.2. Comparative analysis of imaging data

Regarding the foci count, patients in the human fascioliasis group exhibited a significantly lower occurrence of single foci (9.47%) than multiple foci (≥2; 90.53%), while patients in the bacterial liver abscess group primarily exhibited single foci (52.63%). Regarding lesion distribution within the lobes of the liver, human fascioliasis lesions were primarily clustered in the left and right lobes (78.95%). However, bacterial liver abscess predominantly occurred in the right liver lobe (81.05%; *P *< .001). Regarding specific imaging manifestations, patients in the human fascioliasis group exhibited relatively lower rates of complications, including air and liquid cavity formation, ascites, and pleural effusion, at 5.26%, 6.32%, and 8.42%, respectively, than patients in the bacterial liver abscess group (16.84%, 24.21%, and 42.11%, respectively: *P *< .05). No statistically significant difference was observed in the detection of splenomegaly between the 2 groups (Table 2).

**Table 2:** Radiographic data (chest and abdominal CT scans) of patients with human fascioliasis and bacterial liver abscess.

Variable	Human fascioliasis (n = 95)	Bacterial liver abscess (n = 95)	χ^2^	*P*-value
Foci numbers				
Single (n, %)	9 (9.47%)	68 (71.58%)	76.013	<.001
≥2 (n, %)	86 (90.53%)	27 (28.42%)	76.013	<.001
Location of lesion (n, %)				
The right hepatic lobe	11 (5.26%)	77 (81.05%)	92.206	<.001
The left hepatic lobe	9 (9.47%)	12 (12.63%)	0.482	.488
The left and right hepatic lobe	75 (78.95%)	5 (5.26%)	105.795	<.001
Gas cavity formation (n, %)	5 (5.26%)	16 (16.84%)	6.478	.011
Peritoneal effusion (n, %)	6 (6.32%)	23 (24.21%)	11.761	.001
Pleural effusion (n, %)	8 (8.42%)	40 (42.11%)	28.545	<.001
Splenomegaly (n, %)	10 (10.53%)	9 (9.47%)	0.058	.809

Abbreviation: CT: computed tomography.

### 3.3. Analysis of differences in laboratory indicators

Patients in the human fascioliasis group demonstrated significantly lower WBC, N%, TBI, ALT, AST, ALP, GGT, and FIB levels and higher E%, E#, RBC, TP, ALB, CA-125, CA-199, and CA-724 levels than patients in the bacterial liver abscess group. Patients in the human fascioliasis group exhibited a longer thrombin time than those in the bacterial liver abscess group (*P *< .05). No statistically significant difference was observed in other laboratory markers (Table 3).

**Table 3:** Laboratory indicators of patients with human fascioliasis and bacterial liver abscess.

Laboratory indicators	Human fascioliasis (n = 95)	Bacterial liver abscess (n = 95)	χ^2^/*t*/*z*	*P*-value
Blood routine				
White blood cells (×10^9^/L [median, IQR])	8.54 (6.17–12.14)	11.09 (7.81–14.10)	–3.625	<.001
Proportion of neutrophils (% [median, IQR])	38.30 (30.15–47.6)	77.50 (70.75–84.80)	–10.948	<.001
Proportion of eosinophils (% [median, IQR])	30.70 (17.05–43.55)	0.30 (0.10–1.10)	–11.238	<.001
Eosinophil count (×10^9^/L [median, IQR])	2.36 (0.97–4.89)	0.03 (0.01–0.11)	–10.942	<.001
Red blood cells (×10^12^/L [median, IQR])	4.47 (3.99–4.73)	3.91 (3.49–4.45)	–4.030	<.001
Hemoglobin (g/L [mean ± SD])	126.01 ± 20.41	117.13 ± 22.21	–2.87	.005
Platelet (×10^9^/L [median, IQR])	244.00 (198.50–313.00)	255.00 (181.50–393.50)	–0.912	.362
Liver function				
Total bilirubin (μmol/L [median, IQR])	9.60 (7.05–12.75)	11.60 (8.65–16.85)	–3.239	.001
Alanine aminotransferase (U/L [median, IQR])	24.00 (18.00–50.00)	34.00 (21.00–64.50)	–2.520	.012
Aspartate aminotransferase (U/L [median, IQR])	24.00 (18.00–34.50)	32.00 (21.00–56.50)	–3.198	.001
Alkaline phosphatase (U/L [median, IQR])	142.00 (98.00–197.00)	205.00 (123.00–281.00)	–3.970	<.001
Gamma-glutamyl transpeptidase (U/L [median, IQR])	78.00 (44.50–130.50)	179.00 (142.00–241.00)	–6.914	<.001
Globulin (g/L [median, IQR])	33.50 (28.70–40.55)	33.50 (27.65–38.10)	-1.107	.268
Total protein (g/L [median, IQR])	70.00 (66.70–75.60)	60.30 (56.30–69.10)	–6.260	<.001
Albumin (g/L [median, IQR])	37.5 (33.25–41.2)	31.00 (26.50–35.10)	–6.554	<.001
Tumor markers				
Alpha fetoprotein (ng/L [median, IQR])	3.62 (2.29–5.56)	3.25 (2.59–4.87)	–0.427	.427
Carcinoembryonic antigen (ng/L [median, IQR])	2.52 (1.24–5.06)	1.92 (0.92–3.70)	–1.389	.165
Carbohydrate antigen-125 (U/mL [median, IQR])	15.26 (9.99–18.68)	10.32 (5.68–20.27)	–2.588	.010
Carbohydrate antigen 199 (U/mL [median, IQR])	15.82 (4.93–22.86)	4.53 (1.92–11.65)	–4.186	<.001
Carbohydrate antigen 724 (U/mL [median, IQR])	2.03 (1.05–3.69)	1.08 (0.81–2.29)	–3.662	<.001
Coagulation function				
Prothrombin time (s [median, IQR])	12.80 (11.85–13.85)	12.60 (11.75–13.90)	–0.310	.756
Activated partial thromboplastin time (s [median, IQR])	32.60 (26.00–38.25)	31.50 (26.35–38.25)	–0.466	.641
Thrombin time (s [mean ± SD])	17.67 ± 1.46	16.43 ± 1.58	–5.61	<.001
Fibrinogen (g/L [median, IQR])	3.43 (2.85–4.50)	6.33 (5.10–7.81)	–9.314	<.001

Abbreviations: IQR: interquartile range; SD: standard deviation.

The baseline data used in this study were consistent with data in our previously published study.^[10]^

### 3.4. Comparative seasonal distribution of morbidity

**Figure 1.**
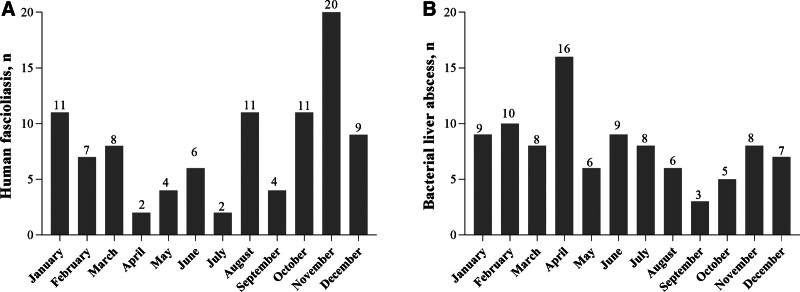
Seasonal onset of human fascioliasis and bacterial liver abscess. The baseline data used in this study were consistent with those of our previously published study.^[10]^

Human fascioliasis peaks during the summer and fall seasons, while it remains relatively low in the spring and winter. However, the incidence of bacterial liver abscess exhibited a more consistent distribution throughout the year (Fig. 1).

## 4. Discussion

This study compared 95 patients with human fascioliasis to 95 patients with bacterial liver abscess to identify differences in baseline characteristics, clinical presentations, imaging findings, laboratory parameters, and seasonal patterns of disease onset. This comprehensive analysis aimed to assist clinicians in differentiating between these 2 conditions early, facilitating more accurate diagnosis and prompt treatment.

Patients with human fascioliasis are generally younger, attributed to unhealthy lifestyle habits and dietary choices among the youth, particularly the consumption of raw food or water contaminated with parasitic eggs.^[11–13]^ Conversely, patients with bacterial liver abscess typically exhibit a higher median age, indicating that diminished physical fitness, compromised immunity, and the existence of chronic underlying conditions may increase disease risk with advancing age. A previous study reported that bacterial liver abscess primarily affects middle-aged and elderly individuals.^[14]^ This study found that females have a higher susceptibility to fascioliasis, consistent with the findings of our previous research.^[10]^ Besides, a study by Leerapun et al,^[6]^ which included 175 patients with fascioliasis, reported that 72% of the patients were female. Furthermore, another study by Chen et al^[15]^ corroborates this finding. One possible explanation is that patients in this region primarily come from rural areas, where most men are employed externally, while women primarily assume responsibilities related to livestock herding and agricultural work. This gender-based division of labor increases the risk of fascioliasis infection in women. The prevalence of bacterial liver abscess is higher in males. This disparity may be ascribed to the modulatory effect of estrogens on immune cells, resulting in females exhibiting enhanced innate immunity and consequently being less susceptible to infection. Previous studies have reported that males are more vulnerable to liver abscess.^[14,16–19]^

Patients with bacterial liver abscess exhibited a higher prevalence of concomitant diabetes mellitus and bile duct stones than those with human fascioliasis. This may be attributed to the fact that patients with diabetes often have a compromised immune system and impaired host defense mechanisms.^[20]^ Moreover, a hyperglycemic state can inhibit the chemotaxis and phagocytosis of leukocytes, thereby creating conditions for bacterial growth. Chronic hyperglycemia can impair the vascular wall, resulting in localized circulatory dysfunction and diminished bacterial clearance by the body,^[21,22]^ consequently increasing the risk of bacterial liver abscess. Diabetes mellitus is recognized as a risk factor for bacterial liver abscess.^[23–25]^ However, patients with human fascioliasis exhibited a lower incidence of biliary stones than those with bacterial liver abscess. Previous studies^[26,27]^ reported that biliary stones are significantly associated with bacterial liver abscess because their obstruction of the biliary system triggers inflammation that retrogresses the liver and culminates in liver abscess. Although disparities in blood pressure readings existed between the 2 groups, no significant difference was observed in the prevalence of underlying hypertensive disease. This finding is inconsistent with those of previous studies^[28]^ that reported that hypertension is a risk factor for bacterial liver abscess. This discrepancy may be attributed to the relatively small sample size of this study, which was conducted at a single center. The common symptom of human fascioliasis is abdominal pain, followed by fever. The fever is believed to be associated with the mechanical injury caused by *Fasciola boydii* in the abdominal cavity and the ensuing inflammatory response in the body. Most patients with human fascioliasis are young individuals with relatively robust immune systems. However, patients with bacterial liver abscess present with fever accompanied by abdominal pain. This may be attributed to the fact that most patients with bacterial liver abscess are elderly patients with diabetes, who often have a higher pain threshold and are susceptible to diabetic peripheral neuropathy. Furthermore, hospitalization duration for patients with fascioliasis is shorter than that for patients with bacterial liver abscess. This is probably because human fascioliasis is a parasitic infection, primarily treated with a complete course of deworming, and its symptoms are less severe.^[10]^ However, a higher proportion of patients with bacterial liver abscess experience abdominal effusion and secondary abdominal infection, leading to prolonged hospitalization.

Imaging reveals that patients with fascioliasis exhibit a significantly higher incidence of multiple foci than those with bacterial liver abscess. This can be attributed to the migratory and reproductive characteristics of the parasites within the liver, resulting in simultaneous or sequential parasite infestations in multiple locations.^[10]^ Bacterial liver abscess are more likely to present as a single focus, indicative of the usual infection source and the mechanism of hematogenous spread to specific liver regions, resulting in localized inflammatory abscess. Human fascioliasis lesions are primarily concentrated in the right and left lobes of the liver, indicating a relatively uniform distribution across all lobes. Conversely, Patients with bacterial liver abscess frequently exhibit significant involvement of the right liver lobe. This suggests that the right lobe of the liver may be more vulnerable to bacterial infections due to anatomical features such as blood flow distribution, as the right lobe is larger.^[24,29]^ Patients with bacterial liver abscess have a higher incidence of pleural and abdominal effusions and gas accumulation within the abscess cavity than those with lamellar fascioliasis. This may be related to the frequent occurrence of fever in patients with bacterial liver abscess, which, combined with the disease process, leads to elevated metabolic consumption and subsequent pleural and abdominal effusions.^[30]^ Gas formation in the abscess cavity is frequently associated with highly virulent gas-producing bacterial infections, including those caused by *Klebsiella pneumoniae*, which are more common in these patients.

Laboratory investigations revealed that WBC, N%, TBI, ALT, AST ALP, and GGT, among other indicators, were significantly lower in patients with human fascioliasis than in those with bacterial liver abscess. Conversely, E%, E#, RBC, HGB, and ALB levels were significantly higher in patients with human fascioliasis than in those with bacterial liver abscess. These alterations may be attributed to the immune response elicited by parasitic infection (*fasciola*). In bacterial liver abscess, activated neutrophils phagocytize pathogens and secrete cytokines to stimulate immune cells. However, an excessive increase in cytokines can disrupt the balance of the immune system, triggering a systemic inflammatory response syndrome and potentially resulting in multiple organ dysfunction. Many patients in the bacterial hepatic abscess group presented with ascites or pleural effusion but exhibited lower CA-125 levels. This may be due to the treatment of bacterial hepatic abscess, which generally includes antibiotics and abscess drainage; therapeutic interventions may reduce CA-125 levels. The relatively small sample size in this study may result in potential data bias. The increased CA-125 and CA-199 levels in patients with human fascioliasis may result from data bias from the relatively small sample size. Furthermore, multicenter studies with larger sample sizes are required to investigate the underlying relationship.

Human fascioliasis demonstrates a clear seasonal distribution, peaking in summer and fall. This pattern is closely associated with the lifecycle of the parasite and the prevailing environmental conditions.^[10]^ The onset of bacterial liver abscess is relatively consistent throughout the year. This uniformity may indicate a diminished correlation with external environmental factors such as temperature and humidity and a stronger influence from individual immunity and medical interventions.

### 4.1. Limitations

This study has some limitations. First, given its retrospective design, the study faced challenges, including selection bias and potential information gaps, which may hinder a comprehensive understanding of all cases, particularly rare and complex ones. Second, our diagnostic approach primarily relied on historical records and pathological data, overlooking the potential of modern molecular biology detection techniques, thereby limiting our ability to comprehensively examine pathogenesis. Third, while we focused on common comorbidities, including diabetes, biliary stones, and hypertension, we did not investigate other potential underlying conditions that might affect disease progression or prognosis. Fourth, the causal relationship between these comorbidities and their specific mechanisms across different disease states was unexamined. Although helpful in identifying typical characteristics, imaging techniques may inadequately differentiate between the 2 diseases when lesions are small, boundaries are indistinct, or overlap with other liver diseases, especially without advanced techniques such as MRI-enhanced scanning and functional imaging. We observed elevated levels of tumor markers CA-199 and CA-724 in human *fascioliasis*; however, the exact causes and clinical significance of these elevations are unclear. The efficacy of these markers as biomarkers for evaluating disease prognosis remains unclear. Fifth, although we observed a distinct seasonal distribution pattern in human fascioliasis, we did not explore the complex relationship between environmental factors, lifestyles, and disease prevalence, nor the effects of climate change on the lifecycle and transmission mechanism of parasites.

## 5. Conclusion

This study has significantly enhanced our understanding of the differences and similarities between human fascioliasis and bacterial liver abscess through comprehensive and meticulous data analysis. This analysis offers robust support for clinical differential diagnosis. Future scientific research can investigate the underlying pathophysiological mechanisms of these 2 diseases, and by integrating these insights with epidemiological features, we can enhance and improve preventive measures and diagnostic and therapeutic protocols. In cases with atypical characteristics, the integration of multiple tests is essential in enhancing the accuracy and promptness of early diagnosis.

Given the nonspecific nature of the clinical and epidemiologic manifestations of human fascioliasis and bacterial liver abscess, it is imperative to exercise caution in differentiating between these 2 diseases in clinical settings.

## Acknowledgments

This study was supported by grants from the subcenter of Clinical Medical Center for Infectious Diseases in Yunnan Province, the construction of Key Laboratory for Infectious Diseases funded by Yunnan Provincial Department of Education, research fund projects awarded by Yunnan Provincial Department of Education (Nos.: 2021J0384 and 2022J0694), a fund project granted by Dali Science and Technology Bureau (No.: 2021KBG037), a key project of discipline development from Dali University (No.: DFYZD2022-06), and the Dali University Discipline Development Backbone Project (No.: DFYGG2022-19).

## Author contributions

**Data curation:** Wei Gu.

**Funding acquisition:** Wei Gu.

**Methodology:** Jiao Luo.

**Resources:** Nan Li.

**Software:** Zhenzhong Bao.

**Writing – original draft:** Lihua Huang.

**Writing – review & editing:** Wei Gu.
